# Supplementary material for: Discovery and application of insertion-deletion (INDEL) polymorphisms for QTL mapping of early life-history traits in Atlantic salmon
Source: BMC Genomics. 2010 Mar 8;11:156. doi: 10.1186/1471-2164-11-156 (PMC2838853; doi:10.1186/1471-2164-11-156)
Supplement: Additional file 2 — Information on developed 76 locus single-run INDEL panel in Atlantic salmon. Information on fluorescence labeling, primer concentrations, PCR pooling and links to alignments, INDEL motifs and GENESCAN (Burge and Karlin 1997) predictions of genes/exons are available in html format. [file 1471-2164-11-156-S2.ZIP › Additionalfile2/table8604.html]

Contig Alignment

```
Base	A B C D E F G H I J K L M N O P Q R S T U V   
1	. . . . A . . . . . A A . . . . . . . . . .    
2	. . . . A . . . . . A A . . . . . . . . . .    
3	. . . . T . . . . . T T . . . . . . . . . .    
4	. . . . C . . C C . C C . . . . . . . . . .    
5	. . . . A . . A A . A A . . . . . . . . . .    
6	. . . . C . . C C C C C . . . . . . . . . .    
7	. . . . A . . A A A A A . . . . . . . . . .    
8	. . . . A . . A A A A A . . . . . . . . . .    
9	. . . . A . . A A A A A . . . . . . . . . .    
10	. . . . A . . A A A A A . . . . . . . . . .    
11	. . . . A . . A A A A A . . . . . . . . . .    
12	. . . . G . . G G G G G . . . . . . . . . .    
13	. . . . A . . A A A A A . . . . . . . . . .    
14	. . . . A . . A A A A A . . . . . . . . . .    
15	. . . . A . . A A A A A . . . . . . . . . .    
16	. . . . A . . A A A A A . . . . . . . . . .    
17	. . . . A . . A A A A A . . . . . . . . . .    
18	. . . . C . . C C C C C . . . . . . . . . .    
19	. . . . T . . T T T T T . . . . . . . . . .    
20	. . . . A . . A A A A A . . . . . . . . . .    
21	. . . . T . . T T T T T . . . . . . . . . .    
22	. . . . G . . G G G G G . . . . . . . . . .    
23	. . . . T . . T T T T T . . . . . . . . . .    
24	. . . . A . . A A A A A . . . . . . . . . .    
25	. . . . T . . T T T T T . . . . . . . . . .    
26	. . . . T . . T T T T T . . . . . . . . . .    
27	. . . . T . . T T T T T . . . . . . . . . .    
28	. . . . A . . A A A A A . . . . . . . . . .    
29	. . . . T . . T T T T T . . . . . . . . . .    
30	. . . . T . . T T T T T . . . . . . . . . .    
31	. . . . A . . A A A A A . . . . . . . . . .    
32	. . . . T . . T T T T T . . . . . . . . . .    
33	. . . . A . . A A A A A . . . . . . . . . .    
34	. . . . A . . A A A A A . . . . . . . . . .    
35	. . . . T . . T T T T T . . . . . . . . . .    
36	. . . . A . . A A A A A . . . . . . . . . .    
37	. . . . A . . A A A A A . . . . . . . . . .    
38	. . . . C . . C C C C C . . . . . . . . . .    
39	. . . . A . . A A A A A . . . . . . . . . .    
40	. . . . G . . G G G G G . . . . . . . . . .    
41	. . . . C . . C C C C C . . . . . . . . . .    
42	. . . . T . . T T T T T . . . . . . . . . .    
43	. . . . A . . A A A A A . . . . . . . . . .    
44	. . . . C . . C C C C C . . . . . . . . . .    
45	A A A A A . . A A A A A A A A A A A A A A A    
46	G G G G G . . G G G G G G G G G G G G G G G    
47	A A A A A . . A A A A A A A A A A A A A A A    
48	A A A A A . . A A A A A A A A A A A A A A A    
49	G G G G G . . G G G G G G G G G G G G G G G    
50	T T T T T . . T T T T T T T T T T T T T T T    
51	C C C C C . . C C C C C C C C C C C C C C C    
52	A A A A A . . A A A A A A A A A A A A A A A    
53	C C C C C . . C C C C C C C C C C C C C C C    
54	A A A A A . . A A A A A A A A A A A A A A A    
55	C C C C C . . C C C C C C C C C C C C C C C    
56	T T T T T . . T T T T T T T T T T T T T T T    
57	A A A A A . . A A A A A A A A A A A A A A A    
58	A A A A A . . A A A A A A A A A A A A A A A    
59	C C C C C . . C C C C C C C C C C C C C C C    
60	A A A A A . . A A A A A A A A A A A A A A A    
61	A A A A A . . A A A A A A A A A A A A A A A    
62	G G G G G . . G G G G G G G G G G G G G G G    
63	C C C C C . . C C C C C C C C C C C C C C C    
64	A A A A A . . A A A A A A A A A A A A A A A    
65	G G G G G . . G G G G G G G G G G G G G G G    
66	C C C C C . . C C C C C C C C C C C C C C C    
67	T T T T T . . T T T T T T T T T T T T T T T    
68	G G G G G . . G G G G G G G G G G G G G G G    
69	C C C C C . . C C C C C C C C C C C C C C C    
70	A A A A A . . A A A A A A A A A A A A A A A    
71	T T T T T . . T T T T T T T T T T T T T T T    
72	A A A A A . . A A A A A A A A A A A A A A A    
73	C C C C C . . C C C C C C C C C C C C C C C    
74	A A A A A . . A A A A A A A A A A A A A A A    
75	A A A A A . . A A A A A A A A A A A A A A A    
76	T T T T T . . T T T T T T T T T T T T T T T    
77	A A A A A . . A A A A A A A A A A A A A A A    
78	A A A A A . . A A A A A A A A A A A A A A A    
79	G G G G G . . G G G G G G G G G G G G G G G    
80	T T T T T . . T T T T T T T T T T T T T T T    
81	A A A A A . . A A A A A A A A A A A A A A A    
82	C C C C C . . C C C C C C C C C C C C C C C    
83	A A A A A . . A A A A A A A A A A A A A A A    
84	A A A A A . . A A A A A A A A A A A A A A A    
85	A A A A A . . A A A A A A A A A A A A A A A    
86	G G G G G . . G G G G G G G G G G G G G G G    
87	G G G G G . . G G G G G G G G G G G G G G G    
88	A A A A A . . A A A A A A A A A A A A A A A    
89	C C C C C . . C C C C C C C C C C C C C C C    
90	A A A A A . . A A A A A A A A A A A A A A A    
91	A A A A A . . A A A A A A A A A A A A A A A    
92	T T T T T . . T T T T T T T T T T T T T T T    
93	A A A A A . . A A A A A A A A A A A A A A A    
94	A A A A A . . A A A A A A A A A A A A A A A    
95	G G G G G . . G G G G G G G G G G G G G G G    
96	T T T T T . . T T T T T T T T T T T T T T T    
97	C C C C C . . C C C C C C C C C C C C C C C    
98	A A A A A . . A A A A A A A A A A A A A A A    
99	T T T T T . . T T T T T T T T T T T T T T T    
100	T T T T T . . T T T T T T T T T T T T T T T    
101	G G G G G . . G G G G G G G G G G G G G G G    
102	G G G G G . . G G G G G G G G G G G G G G G    
103	A A A A A . . A A A A A A A A A A A A A A A    
104	T T T T T . . T T T T T T T T T T T T T T T    
105	T T T T T . . T T T T T T T T T T T T T T T    
106	T T T T T . . T T T T T T T T T T T T T T T    
107	A A A A A . . A A A A A A A A A A A A A A A    
108	T T T T T . . T T T T T T T T T T T T T T T    
109	G G G G G . . G G G G G G G G G G G G G G G    
110	G G G G G . . G G G G G G G G G G G G G G G    
111	C C C C C . . C C C C C C C C C C C C C C C    
112	G G G G G . . G G G G G G G G G G G G G G G    
113	T T T T T . . T T T T T T T T T T T T T T T    
114	C C C C C . . C C C C C C C C C C C C C C C    
115	A A A A A . . A A A A A A A A A A A A A A A    
116	T T T T T . . T T T T T T T T T T T T T T T    
117	T T T T T . . T T T T T T T T T T T T T T T    
118	G G G G G . . G G G G G G G G G G G G G G G    
119	T T T T T . . T T T T T T T T T T T T T T T    
120	T T T T T . . T T T T T T T T T T T T T T T    
121	G G G G G . . G G G G G G G G G G G G G G G    
122	G G G G G . . G G G G G G G G G G G G G G G    
123	G G G G G . . G G G G G G G G G G G G G G G    
124	T T T T T . . T T T T T T T T T T T T T T T    
125	G G G G G . . G G G G G G G G G G G G G G G    
126	A A A A A . . A A A A A A A A A A A A A A A    
127	G G G G G . . G G G G G G G G G G G G G G G    
128	T T T T T . . T T T T T T T T T T T T T T T    
129	A A A A A . . A A A A A A A A A A A A A A A    
130	G G G G G . . G G G G G G G G G G G G G G G    
131	C C C C C . . C C C C C C C C C C C C C C C    
132	A A A A A . . A A A A A A A A A A A A A A A    
133	A A A A A . . A A A A A A A A A A A A A A A    
134	A A A A A . . A A A A A A A A A A A A A A A    
135	G G G G G . . G G G G G G G G G G G G G G G    
136	C C C C C . . C C C C C C C C C C C C C C C    
137	T T T T T . . T T T T T T T T T T T T T T T    
138	C C C C C . . C C C C C C C C C C C C C C C    
139	T T T T T . . T T T T T T T T T T T T T T T    
140	G G G G G . . G G G G G G G G G G G G G G G    
141	C C C C C . . C C C C C C C C C C C C C C C    
142	G G G G G . . G G G G G G G G G G G G G G G    
143	G G G G G . . G G G G G G G G G G G G G G G    
144	C C C C C . . C C C C C C C C C C C C C C C    
145	T T T T T . . T T T T T T T T T T T T T T T    
146	G G G G G . . G G G G G G G G G G G G G G G    
147	G G G G G . . G G G G G G G G G G G G G G G    
148	A A A A A . . A A A A A A A A A A A A A A A    
149	T T T T T . . T T T T T T T T T T T T T T T    
150	T T T T T . . T T T T T T T T T T T T T T T    
151	C C C C C . . C C C C C C C C C C C C C C C    
152	A A A A A . . A A A A A A A A A A A A A A A    
153	G G G G G . . G G G G G G G G G G G G G G G    
154	T T T T T . . T T T T T T T T T T T T T T T    
155	G G G G G . . G G G G G G G G G G G G G G G    
156	C C C C C . . C C C C C C C C C C C C C C C    
157	T T T T T . . T T T T T T T T T T T T T T T    
158	T T T T T . . T T T T T T T T T T T T T T T    
159	T T T T T . . T T T T T T T T T T T T T T T    
160	T T T T T . . T T T T T T T T T T T T T T T    
161	C C C C C . . C C C C C C C C C C C C C C C    
162	T T T T T . . T T T T T T T T T T T T T T T    
163	T T T T T . . T T T T T T T T T T T T T T T    
164	T T T T T . . T T T T T T T T T T T T T T T    
165	C C C C C . . C C C C C C C C C C C C C C C    
166	T T T T T . . T T T T T T T T T T T T T T T    
167	C C C C C . . C C C C C C C C C C C C C C C    
168	C C C C C . . C C C C C C C C C C C C C C C    
169	C C C C C . . C C C C C C C C C C C C C C C    
170	T T T T T . . T T T T T T T T T T T T T T T    
171	G G G G G . . G G G G G G G G G G G G G G G    
172	T T T T T . . T T T T T T T T T T T T T T T    
173	G G G G G . . G G G G G G G G G G G G G G G    
174	T T T T T . . T T T T T T T T T T T T T T T    
175	G G G G G . . G G G G G G G G G G G G G G G    
176	- - - - T . . T T T T T - - - - - - - - - -  6	 indel  
177	- - - - G . . G G G G G - - - - - - - - - -  6	 indel  
178	- - - - T . . T T T T T - - - - - - - - - -  6	 indel  
179	- - - - G . . G G G G G - - - - - - - - - -  6	 indel  
180	- - - - T . . T T T T T - - - - - - - - - -  6	 indel  
181	- - - - G . . G G G G G - - - - - - - - - -  6	 indel  
182	C C C C C . . C C C C C C C C C C C C C C C    
183	G G G G G . . G G G G G G G G G G G G G G G    
184	T T T T T . . T T T T T T T T T T T T T T T    
185	G G G G G . . G G G G G G G G G G G G G G G    
186	T T T T T . . T T T T T T T T T T T T T T T    
187	G G G G G . . G G G G G G G G G G G G G G G    
188	C C C C C . . C C C C C C C C C C C C C C C    
189	G G G G G . . G G G G G G G G G G G G G G G    
190	T T T T T . . T T T T T T T T T T T T T T T    
191	C C C C C . . C C C C C C C C C C C C C C C    
192	C C C C C . . C C C C C C C C C C C C C C C    
193	A A A A A . . A A A A A A A A A A A A A A A    
194	T T T T T . . T T T T T T T T T T T T T T T    
195	G G G G G . . G G G G G G G G G G G G G G G    
196	C C C C C . . C C C C C C C C C C C C C C C    
197	C C C C C . . C C C C C C C C C C C C C C C    
198	A A A A A . . A A A A A A A G G A A A A A A    
199	T T T T T . . T T T T T T T T T T T T T T T    
200	C C C C C . . C C C C C C C C C C C C C C C    
201	T T T T T . . T T T T T T T T T T T T T T T    
202	G G G G G . . G G G G G G G G G G G G G G G    
203	C C C C C . . C C C C C C C C C C C C C C C    
204	T T T T T . . T T T T T T T T T T T T T T T    
205	G G G G G . . G G G G G G G G G G G G G G G    
206	G G G G G . . G G G G G G G G G G G G G G G    
207	T T T T T . . T T T T T T T T T T T T T T T    
208	C C C C C . . C C C C C C C C C C C C C C C    
209	A A A A A . . A A A A A A A A A A A A A A A    
210	G G G G G . . G G G G G G G G G G G G G G G    
211	A A A A A . . A A A A A A A A A A A A A A A    
212	C C C C C . . C C C C C C C C C C C C C C C    
213	C C C C C . . C C C C C C C C C C C C C C C    
214	T T T T T . . T T T T T T T T T T T T T T T    
215	T T T T T . . T T T T T T T T T T T T T T T    
216	G G G G G . . G G G G G G G G G G G G G G G    
217	C C C C C . . C C C C C C C C C C C C C C C    
218	C C C C C . . C C C C C C C C C C C C C C C    
219	C C C C C . . C C C C C C C C C C C C C C C    
220	A A A A A . . A A A A A A A A A A A A A A A    
221	C C C C C . . C C C C C C C C C C C C C C C    
222	A A A A A . . A A A A A A A A A A A T T A A    
223	G G G G G . . G G G G G G G G G G G G G G G    
224	C C C C C . . C C C C C C C C C C C C C C C    
225	C C C C C . . C C C C C C C C C C C C C C C    
226	A A A A A . . A A A A A A A A A A A A A A A    
227	G G G G G . . G G G G G G G G G G G G G G G    
228	G G G G G . . G G G G G G G G G G G G G G G    
229	C C C C C . . C C C C C C C C C C C C C C C    
230	C C C C C . . C C C C C C C C C C C C C C C    
231	C C C C C . . C C C C C C C C C C C C C C C    
232	T T T T T . . T T T T T T T T T T T T T T T    
233	G G G G G . . G G G G G G G G G G G G G G G    
234	A A A A A . . A A A A A A A A A A A A A A A    
235	G G G G G . . G G G G G G G G G G G G G G G    
236	C C C C C . . C C C C C C C C C C C C C C C    
237	C C C C C . . C C C C C C C C C C C C C C C    
238	C C C C C . . C C C C C C C C C C C C C C C    
239	T T T T T . . T T T T T T T T T T T T T T T    
240	C C C C C . . C C C C C C C C C C C C C C C    
241	T T T T T . . T T T T T T T T T T T T T T T    
242	G G G G G . G G G G G G G G G G G G G G G G    
243	G G G G G . G G G G G G G G G G G G G G G G    
244	T T T T T . T T T T T T T T T T T T T T T T    
245	A A A A A . A A A A A A A A A A A A A A A A    
246	C C C C C . C C C C C C C C C C C C C C C C    
247	T T T T T . T T T T T T T T T T T T T T T T    
248	C C C C C . C C C C C C C C C C C C C C C C    
249	T T T T T . T T T T T T T T T T T T T T T T    
250	T T T T T . T T T T T T T T T T T T T T T T    
251	G G G G G . G G G G G G G G G G G G G G G G    
252	C C C C C . C C C C C C C C C C C C C C C C    
253	T T T T T . T T T T T T T T T T T T T T T T    
254	G G G G G . G G G G G G G G G G G G G G G G    
255	C C C C C . C C C C C C C C C C C C C C C C    
256	A A A A A . A A A A A A A A A A A A A A A A    
257	G G G G G . G G G G G G G G G G G G G G G G    
258	A A A A A . A A A A A A A A A A A A A A A A    
259	C C C C C . C C C C C C C C C C C C C C C C    
260	T T T T T . T T T T T T T T T T T T T T T T    
261	A A A A A . A A A A A A A A A A A A A A A A    
262	G G G G G G G G G G G G G G G G G G G G G G    
263	A A A A A A A A A A A A A A A A A A A A A A    
264	C C C C C C C C C C C C C C C C C C C C C C    
265	A A A A A A A A A A A A A A A A A A A A A A    
266	G G G G G G G G G G G G G G G G G G G G G G    
267	G G G G G G G G G G G G G G G G G G G G G G    
268	A A A A A A A A A A A A A A A A A A A A A A    
269	C C C C C C C C C C C C C C C C C C C C C C    
270	C C C C C C C C C C C C C C C C C C C C C C    
271	T T T T T T T T T T T T T T T T T T T T T T    
272	C C C C C C C C C C C C C C C C C C C C C C    
273	A A A A A A A A A A A A A A A A A A A A A A    
274	C C C C C C C C C C C C C C C C C C C C C C    
275	G G G G G G G G G G G G G G G G G G G G G G    
276	G G G G G G G G G G G G G G G G G G G G G G    
277	T G T T T T T T T T T T T T T T T T C C T T    
278	G G G G G G G G G G G G G G G G G G G G G G    
279	C C C C C C C C C C C C C C C C C C C C C C    
280	T T T T T T T T T T T T T T T T T T T T T T    
281	G G G G G G G G G G G G G G G G G G G G G G    
282	A A A A A A A A A A A A A A A A A A A A A A    
283	T T T T T T T T T T T T T T T T T T T T T T    
284	C C C C C C C C C C C C C C C C C C C C C C    
285	A A A A A A A A A A A A A A A A A A A A A A    
286	G G G G G G G G G G G G G G G G G G G G G G    
287	A A A A A A A A A A A A A A A A A A A A A A    
288	C C C C C C C C C C C C C C C C C C C C C C    
289	T T T T T T T T T T T T T T T T T T T T T T    
290	T T T T T T T T T T T T T T T T T T T T T T    
291	C C C C C C C C C C C C C C C C C C C C C C    
292	T T T T T T T T T T T T T T T T T T T T T T    
293	T T T T T T T T T T T T T T T T T T T T T T    
294	C C C C C C C C C C C C C C C C C C C C C C    
295	T T T T T T T T T T T T T T T T T T T T T T    
296	C C C C C C C C C C C C C C C C C C C C C C    
297	C C C C C C C C C C C C C C C C C C C C C C    
298	A A A A A A A A A A A A A A A A A A A A A A    
299	T T T T T T T T T T T T T T T T T T T T T T    
300	G G G G G G G G G G G G G G G G G G G G G G    
301	T T T T T T T T T T T T T T T T T T T T T T    
302	C C C C C C C C C C C C C C C C C C C C C C    
303	C C C C C C C C C C C C C C C C C C C C C C    
304	T T T T T T T T T T T T T T T T T T T T T T    
305	T T T T T T T T T T T T T T T T T T T T T T    
306	T T T T T T T T T T T T T T T T T T T T T T    
307	A A A A A A A A A A A A A A A A A A A A A A    
308	G G G G G G G G G G G G G G G G G G G G G G    
309	T T T T T T T T T T T T T T T T T T T T T T    
310	A A A A A A A A A A A A A A A A A A A A A A    
311	G G G G G G G G G G G G G G G G G G G G G G    
312	A A A A A A A A A A A A A A A A A A A A A A    
313	G G G G G G G G G G G G G G G G G G G G G G    
314	T T T T T T T T T T T T T T T T T T T T T T    
315	C C C C C C C C C C C C C C C C C C C C C C    
316	T T T T T T T T T T T T T T T T T T T T T T    
317	C C C C C C C C C C C C C C C C C C C C C C    
318	G G G G G G G G G G G G G G G G G G G G G G    
319	T T T T T T T T T T T T T T T T T T T T T T    
320	A A A A A A A A A A A A A A A A A A A A A A    
321	T T T T T T T T T T T T T T T T T T T T T T    
322	C C C C C C C C C C C C C C C C C C C C C C    
323	T T T T T T T T T T T T T T T T T T T T T T    
324	T T T T T T T T T T T T T T T T T T T T T T    
325	T T T T T T T T T T T T T T T T T T T T T T    
326	G G G G G G G G G G G G G G G G G G G G G G    
327	A A A A A A A A A A A A A A A A A A A A A A    
328	A A A A A A A A A A A A A A A A A A A A A A    
329	T T T T T T T T T T T T T T T T T T T T T T    
330	C C C C C C C C C C C C C C C C C C C C C C    
331	T T T T T T T T T T T T T T T T T T T T T T    
332	C C C C C C C C C C C C C C C C C C C C C C    
333	C C C C C C C C C C C C C C C C C C C C C C    
334	C C C C C C C C C C C C C C C C C C C C C C    
335	C C C C C C C C C C C C C C C C C C C C C C    
336	A A A A A A A A A A A A A A A A A A A A A A    
337	T T T T T T T T T T T T T T T T T T T T T T    
338	T T T T T T T T T T T T T T T T T T T T T T    
339	T T T T T T T T T T T T T T T T T T T T T T    
340	A A A A A A A A A A A A A A A A A A A A A A    
341	T T T T T T T T T T T T T T T T T T T T T T    
342	G G G G G G G G G G G G G G G G G G G G G G    
343	T T T T T T T T T T T T T T T T T T T T T T    
344	A A A A A A A A A A A A A A A A A A A A A A    
345	T T T T T T T T T T T T T T T T T T T T T T    
346	T T T T T T T T T T T T T T T T T T T T T T    
347	C C C C C C C C C C C C C C C C C C C C C C    
348	T T T T T T T T T T T T T T T T T T T T T T    
349	A A A A A A A A A A A A A A A A A A A A A A    
350	G G G G G G G G G G G G G G G G G G G G G G    
351	C C C C C C C C C C C C C C C C C C C C C C    
352	C C C C C C C C C C C C C C C C C C C C C C    
353	T T T T T T T T T T T T T T T T T T T T T T    
354	C C C C C C C C C C C C C C C C C C C C C C    
355	T T T T T T T T T T T T T T T T T T T T T T    
356	T T T T T T T T T T T T T T T T T T T T T T    
357	T T T T T T T T T T T T T T T T T T T T T T    
358	G G G G G G G G G G G G G G G G G G G G G G    
359	C C C C C C C C C C C C C C C C C C C C C C    
360	C C C C C C C C C C C C C C C C C C C C C C    
361	A A A A A A A A A A A A A A A A A A A A A A    
362	C C C C C C C C C C C C C C C C C C C C C C    
363	T T T T T T T T T T T T T T T T T T T T T T    
364	G G G G G G G G G G G G G G G G G G G G G G    
365	T G T T T T T T T T T T T T T T T T T T T T    
366	G G G G G G G G G G G G G G G G G G G G G G    
367	G G G G G G G G G G G G G G G G G G G G G G    
368	C C C C C C C C C C C C C C C C C C C C C C    
369	T T T T T T T T T T T T T T T T T T T T T T    
370	T T T T T T T T T T T T T T T T T T T T T T    
371	T T T T T T T T T T T T T T T T T T T T T T    
372	G G G G G G G G G G G G G G G G G G G G G G    
373	G G G G G G G G G G G G G G G G G G G G G G    
374	C C C C C C C C C C C C C C C C C C C C C C    
375	T T T T T T T T T T T T T T T T T T T T T T    
376	T T T T T T T T T T T T T T T T T T T T T T    
377	C C C C C C C C C C C C C C C C C C C C C C    
378	C C C C C C C C C C C C C C C C C C C C C C    
379	T T T T T T T T T T T T T T T T T T T T T T    
380	C C C C C C C C C C C C C C C C C C C C C C    
381	C C C C C C C C C C C C C C C C C C C C C C    
382	A A A A A A A A A A A A A A A A A A A A A A    
383	A A A A A A A A A A A A A A A A A A A A A A    
384	G G G G G G G G G G G G G G G G G G G G G G    
385	T T T T T T T T T T T T T T T T T T T T T T    
386	C C C C C C C C C C C C C C C C C C C C C C    
387	T T T T T T T T T T T T T T T T T T T T T T    
388	T T T T T T T T T T T T T T T T T T T T T T    
389	G G G G G G G G G G G G G G G G G G G G G G    
390	C C C C C C C C C C C C C C C C C C C C C C    
391	T T T T T T T T T T T T T T T T T T T T T T    
392	T T T T T T T T T T T T T T T T T T T T T T    
393	C C C C C C C C C C C C C C C C C C C C C C    
394	A A A A A A A A A A A A A A A A A A A A A A    
395	C C C C C C C C C C C C C C C C C C C C C C    
396	C C C C C C C C C C C C C C C C C C C C C C    
397	A A A A A A A A A A A A A A A A A A A A A A    
398	G G G G G G G G G G G G G G G G G G G G G G    
399	A A A A A A A A A A A A A A A A A A A A A A    
400	A A A A A A A A A A A A A A A A A A A A A A    
401	C C C C C C C C C C C C C C C C C C C C C C    
402	T T T T T T T T T T T T T T T T T T T T T T    
403	G G G G G G G G G G G G G G G G G G G G G G    
404	G G G G G G G G G G G G G G G G G G G G G G    
405	A A A A A A A A A A A A A A A A A A A A A A    
406	C C C C C C C C C C C C C C C C C C C C C C    
407	C C C C C C C C C C C C C C C C C C C C C C    
408	T T T T T T T T T T T T T T T T T T T T T T    
409	A A A A A A A A A A A A A A A A A A A A A A    
410	T T T T T T T T T T T T T T T T T T T T T T    
411	A A A A A A A A A A A A A A A A A A A A A A    
412	A A A A A A A A A A A A A A A A A A A A A A    
413	G G G G G G G G G G G G G G G G G G G G G G    
414	C C C C C C C C C C C C C C C C C C C C C C    
415	T T T T T T T T T T T T T T T T T T T T T T    
416	T T T T T T T T T T T T T T T T T T T T T T    
417	G G G G G G G G G G G G G G G G G G G G G G    
418	T T T T T T T T T T T T T T T T T T T T T T    
419	A A A A A A A A A A A A A A A A A A A A A A    
420	A A A A A A A A A A A A A A A A A A A A A A    
421	A A A A A A A A A A A A A A A A A A A A A A    
422	C C C C C C C C C C C C C C C C C C C C C C    
423	T T T T T T T T T T T T T T T T T T T T T T    
424	G G G G G G G G G G G G G G G G G G G G G G    
425	T T T T T T T T T T T T T T T T T T T T T T    
426	G G G G G G G G G G G G G G G G G G G G G G    
427	T T T T T T T T T T T T T T T T T T T T T T    
428	T T T T T T T T T T T T T T T T T T T T T T    
429	C C C C C C C C C C C C C C C C C C C C C C    
430	T T T T T T T T T T T T T T T T T T T T T T    
431	G G G G G G G G G G G G G G G G G G G G G G    
432	G G G G G G G G G G G G G G G G G G G G G G    
433	G G G G G G G G G G G G G G G G G G G G G G    
434	T T T T T T T T T T T T T T T T T T T T T T    
435	G G G G G G G G G G G G G G G G G G G G G G    
436	T T T T T T T T T T T T T T T T T T T T T T    
437	C C C C C C C C C C C C C C C C C C C C C C    
438	C C C C C C C C C C C C C C C C C C C C C C    
439	A A A A A A A A A A A A A A A A A A A A A A    
440	A A A A A A A A A A A A A A A A A A A A A A    
441	C C C C C C C C C C C C C C C C C C C C C C    
442	A A A A A A A A A A A A A A A A A A A A A A    
443	A A A A A A A A A A A A A A A A A A A A A A    
444	G G G G G G G G G G G G G G G G G G G G G G    
445	T T T T T T T T T T T T T T T T T T T T T T    
446	C C C C C C C C C C C C C C C C C C C C C C    
447	C C C C C C C C C C C C C C C C C C C C C C    
448	A A A A A A A A A A A A A A A A A A A A A A    
449	A A A A A A A A A A A A A A A A A A A A A A    
450	C C C C C C C C C C C C C C C C C C C C C C    
451	T T T T T T T T T T T T T T T T T T T T T T    
452	C C C C C C C C C C C C C C C C C C C C C C    
453	C C C C C C C C C C C C C C C C C C C C C C    
454	T T T T T T T T T T T T T T T T T T T T T T    
455	C C C C C C C C C C C C C C C C C C C C C C    
456	T T T T T T T T T T T T T T T T T T T T T T    
457	T T T T T T T T T T T T T T T T T T T T T T    
458	T T T T T T T T T T T T T T T T T T T T T T    
459	G G G G G G G G G G G G G G G G G G G G G G    
460	A A A A A A A A A A A A A A A A A A A A A A    
461	C C C C C C C C C C C C C C C C C C C C C C    
462	T T T T T T T T T T T T T T T T T T T T T T    
463	A A A A A A A A A A A A A A A A A A A A A A    
464	T T T T T T T T T T T T T T T T T T T T T T    
465	A A A A A A A A A A A A A A A A A A A A A A    
466	T T T T T T T T T T T T T T T T T T T T T T    
467	T T T T T T T T T T T T T T T T T T T T T T    
468	A A A A A A A A A A A A A A A A A A A A A A    
469	T T T T T T T T T T T T T T T T T T T T T T    
470	T T T T T T T T T T T T T T T T T T T T T T    
471	C C C C C C C C C C C C C C C C C C C C C C    
472	T T T T T T T T T T T T T T T T T T T T T T    
473	C C C C C C C C C C C C C C C C C C C C C C    
474	T T T T T T T T T T T T T T T T T T T T T T    
475	G G G G G G G G G G G G G G G G G G G G G G    
476	T T T T T T T T T T T T T T T T T T T T T T    
477	C C C C C C C C C C C C C C C C C C C C C C    
478	A A A A A A A A A A A A A A A A A A A A A A    
479	C C C C C C C C C C C C C C C C C C C C C C    
480	C C C C C C C C C C C C C C C C C C C C C C    
481	T T T T T T T T T T T T T T T T T T T T T T    
482	G G G G G G G G G G G G G G G G G G G G G G    
483	A A A A A A A A A A A A A A A A A A A A A A    
484	G G G G G G G G G G G G G G G G G G G G G G    
485	C C C C C C C C C C C C C C C C C C C C C C    
486	C C C C C C C C C C C C C C C C C C C C C C    
487	T T T T T T T T T T T T T T T T T T T T T T    
488	C C C C C C C C C C C C C C C C C C C C C C    
489	C C C C C C C C C C C C C C C C C C C C C C    
490	A A A A A A A A A A A A A A A A A A A A A A    
491	G G G G G G G G G G G G G G G G G G G G G G    
492	T T T T T T T T T T T T T T T T T T T T T T    
493	T T T T T T T T T T T T T T T T T T T T T T    
494	T T T T T T T T T T T T T T T T T T T T T T    
495	C C C C C C C C C C C C C C C C C C C C C C    
496	T T T T T T T T T T T T T T T T T T T T T T    
497	G G G G G G G G G G G G G G G G G G G G G G    
498	C C C C C C C C C C C C C C C C C C C C C C    
499	C C C C C C C C C C C C C C C C C C C C C C    
500	T T T T T T T T T T T T T T T T T T T T T T    
501	G G G G G G G G G G G G G G G G G G G G G G    
502	G G G G G G G G G G G G G G G G G G G G G G    
503	C C C C C C C C C C C C C C C C C C C C C C    
504	T T T T T T T T T T T T T T T T T T T T T T    
505	G G G G G G G G G G G G G G G G G G G G G G    
506	A A A A A A A A A A A A A A A A A A A A A A    
507	C C C C C C C C C C C C C C C C C C C C C C    
508	A A A A A A A A A A A A A A A A A A A A A A    
509	T T T T T T T T T T T T T T T T T T T T T T    
510	G G G G G G G G G G G G G G G G G G G G G G    
511	C C C C C C C C C C C C C C C C C C C C C C    
512	T T T T T T T T T T T T T T T T T T T T T T    
513	C C C C C C C C C C C C C C C C C C C C C C    
514	T T T T T T T T T T T T T T T T T T T T T T    
515	T T T T T T T T T T T T T T T T T T T T T T    
516	A A A A A A A A A A A A A A A A A A A A A A    
517	C C C C C C C C C C C C C C C C C C C C C C    
518	T T T T T T T T T T T T T T T T T T T T T T    
519	A A A A A A A A A A A A A A A A A A A A A A    
520	A A A A A A A A A A A A A A A A A A A A A A    
521	C C C C C C C C C C C C C C C C C C C C C C    
522	A A A A A A A A A A A A A A A A A A A A A A    
523	T T T T T T T T T T T T T T T T T T T T T T    
524	C C C C C C C C C C C C C C C C C C C C C C    
525	T T T T T T T T T T T T T T T T T T T T T T    
526	T T T T T T T T T T T T T T T T T T T T T T    
527	T T T T T T T T T T T T T T T T T T T T T T    
528	T T T T T T T T T T T T T T T T T T T T T T    
529	T T T T T T T T T T T T T T T T T T T T T T    
530	G G G G G G G G G G G G G G G G G G G G G G    
531	C C C C C C C C C C C C C C C C C C C C C C    
532	A A A A A A A A A A A A A A A A A A A A A A    
533	T T T T T T T T T T T T T T T T T T T T T T    
534	C C C C C C C C C C C C C C C C C C C C C C    
535	T T T T T T T T T T T T T T T T T T T T T T    
536	G G G G G G G G G G G G G G G G G G G G G G    
537	C C C C C C C C C C C C C C C C C C C C C C    
538	T T T T T T T T T T T T T T T T T T T T T T    
539	G G G G G G G G G G G G G G G G G G G G G G    
540	G G G G G G G G G G G G G G G G G G G G G G    
541	T T T T T T T T T T T T T T T T T T T T T T    
542	A A A A A A A A A A A A A A A A A A A A A A    
543	T T T T T T T T T T T T T T T T T T T T T T    
544	T T T T T T T T T T T T T T T T T T T T T T    
545	T T T T T T T T T T T T T T T T T T T T T T    
546	T T T T T T T T T T T T T T T T T T T T T T    
547	T T T T T T T T T T T T T T T T T T T T T T    
548	C C C C C C C C C C C C C C C C C C C C C C    
549	T T T T T T T T T T T T T T T T T T T T T T    
550	A A A A A A A A A A A A A A A A A A A A A A    
551	G G G G G G G G G G G G G G G G G G G G G G    
552	C C C C C C C C C C C C C C C C C C C C C C    
553	T T T T T T T T T T T T T T T T T T T T T T    
554	C C C C C C C C C C C C C C C C C C C C C C    
555	C C C C C C C C C C C C C C C C C C C C C C    
556	G G G G G G G G G G G G G G G G G G G G G G    
557	A A A A A A A A A A A A A A A A A A A A A A    
558	T T T T T T T T T T T T T T T T T T T T T T    
559	T T T T T T T T T T T T T T T T T T T T T T    
560	G G G G G G G G G G G G G G G G G G G G G G    
561	T T T T T T T T T T T T T T T T T T T T T T    
562	A A A A A A A A A A A A A A A A A A A A A A    
563	A A A A A A A A A A A A A A A A A A A A A A    
564	T T T T T T T T T T T T T T T T T T T T T T    
565	T T T T T T T T T T T T T T T T T T T T T T    
566	T T T T T T T T T T T T T T T T T T T T T T    
567	C C C C C C C C C C C C C C C C C C C C C C    
568	T T T T T T T T T T T T T T T T T T T T T T    
569	T T T T T T T T T T T T T T T T T T T T T T    
570	C C C C C C C C C C C C C C C C C C C C C C    
571	T T T T T T T T T G T T T T T T T T T T T T    
572	G G G G G G G G G G G G G G G G G G G G G G    
573	A A A A A A A A A A A A A A A A A A A A A A    
574	A A A A A A A A A A A A A A A A A A A A A A    
575	T T T T T T T T T T T T T T T T T T T T T T    
576	C C C C C C C C C C C C C C C C C C C C C C    
577	G G G G G G G G G G G G G G G G G G G G G G    
578	C C C C C C C C C C C C C C C C C C C C C C    
579	C C C C C C C C C C C C C C C C C C C C C C    
580	T T T T T T T T T T T T T T T T T T T T T T    
581	C C C C C C C C C C C C C C C C C C C C C C    
582	T T T T T T T T T T T T T T T T T T T T T T    
583	G G G G G G G G G G G G G G G G G G G G G G    
584	. C C C C C C C C C C C C C C C C C C C C C    
585	. C C C C C C C C C C C C C C C C C C C C C    
586	. A A A A A A A A A A A A A A A A A A A A A    
587	. T T T T T T T T T T T T T T T T T T T T T    
588	. A A A A A A A A A A A A A A A A A A A A A    
589	. T T T T T T T T T T T T T T T T T T T T T    
590	. . C C C C C C C C C C C C C C C C C C C C    
591	. . T T T T T T T T T T T T T T T T T T T T    
592	. . A A A A A A A A A A A A A A A A A A A A    
593	. . C C C C C C C C C C C C C C C C C C C C    
594	. . C G G G G G G G G G G G G G G G G G G G    
595	. . G G G G G G G G G G G G G G G G G G G G    
596	. . T T T T T T T G T T T T T T T T T T T T    
597	. . G G G G G G G G G G G G G G G G G G G G    
598	. . T T T T T T T T T T T T T T T T T T T T    
599	. . T T T T T T T T T T T T T T T T T T T T    
600	. . T T T T T T T T T T T T T T T T T T T T    
601	. . C C C C C C C C C C C C C C C C C C C C    
602	. . T T T T T T T T T T T T T T T T T T T T    
603	. . G G G G G G G G G G G G G G G G G G G G    
604	. . A A A A A A A A A A A A A A A A A A A A    
605	. . T T T T T T T T T T T T T T T T T T T T    
606	. . C C C C C C C C C C C C C C C C C C C C    
607	. . A A A A A A A A A A A A A A A A A A A A    
608	. . A A A A A A A A A A A A A A A A A A A A    
609	. . G G G G G G G G G G G G G G G G G G G G    
610	. . C C C C C C C C C C C C C C C C C C C C    
611	. . C C C C C C C C C C C C C C C C C C C C    
612	. . T T T T T T T T T T T T T T T T T T T T    
613	. . T T T T T T T T T T T T T T T T T T T T    
614	. . T T T T T T T T T T T T T T T T T T T T    
615	. . G G G G G G G G G G G G G G G G G G G G    
616	. . T T T T T T T T T T T T T T T T T T T T    
617	. . T T T T T T T T T T T T T T T T T T T T    
618	. . T T T T T T T T T T T T T T T T T T T T    
619	. . A A A A A A A A A A A A A A A A A A A A    
620	. . T T T T T T T T T T T T T T T T T T T T    
621	. . T T T T T T T T T T T T T T T T T T T T    
622	. . A A A A A A A A A A A A A A A A A A A A    
623	. . T T T T T T T T T T T T T T T T T T T T    
624	. . A A A A A A A A A A A A A A A A A A A A    
625	. . A A A A A A A A A A A A A A A A A A A A    
626	. . A A A A A A A A A A A A A A A A A A A A    
627	. . C C C C C C C C C C C C C C C C C C C C    
628	. . T T T T T T T T T T T T T T T T T T T T    
629	. . G G G G G G G G G G G G G G G G G G G G    
630	. . A A A A A A A A A A A A A A A A A A A A    
631	. . A A A A A A A A A A A A A A A A A A A A    
632	. . A A A A A A A A A A A A A A A A A A A A    
633	. . C C C C C C C C C C C C C C C C C C C C    
634	. . G G G G G G G G G G G G G G G G G G G G    
635	. . A A A A A A A A A A A A A A A A A A A A    
636	. . A A A A A A A A A A A A A A A A A A A A    
637	. . A A A A A A A A A A A A A A A A A A A A    
638	. . T T T T T T T T T T T T T T T T T T T T    
639	. . C C C C C C C C C C C C C C C C C C C C    
640	. . A A A A A A A A A A A A A A A A A A A A    
641	. . G G G G G G G G G G G G G G G G G G G G    
642	. . T T T T T T T T T T T T T T T T T T T T    
643	. . T T T T T T T T T T T T T T T T T T T T    
644	. . C C C C C C C C C C C C C C C C C C C C    
645	. . C C C C C C C C C C C C C C C C C C C C    
646	. . T T T T T T T T T T T T T T T T T T T T    
647	. . A A A A A A A A A A A A A A A A A A A A    
648	. . T T T T T T T T T T T T T T T T T T T T    
649	. . A A A A A A A A A A A A A A A A A A A A    
650	. . C C C C C C C C C C C C C C C C C C C C    
651	. . C C C C C C C C C C C C C C C C C C C C    
652	. . T T T T T T T T T T T T T T T T T T T T    
653	. . . A A A A A A A A A A A A A A A A A A A    
654	. . . G G G G G G G G G G G G G G G G G G G    
655	. . . C C C C C C C C C C C C C C C C C C C    
656	. . . A A A A A A A A A A A A A A A A A A A    
657	. . . G G G G G G G G G G G G G G G G G G G    
658	. . . C C C C C C C C C C C C C C C C C C C    
659	. . . A A A A A A A A A A A A A A A A A A A    
660	. . . . A A A A A A A A A A A A A A A A A A    
661	. . . . C C C C C C C C C C C C C C C C C C    
662	. . . . A A A A A A A A A A A A A A A A A A    
663	. . . . G G G G G G G G G G G G G G G G G G    
664	. . . . A A A A A A A A A A A A A A A A A A    
665	. . . . A A A A A A A A A A A A A A A A A A    
666	. . . . T T T T T T T T T T T T T T T T T T    
667	. . . . A A A A A A A A A A A A A A A A A A    
668	. . . . T T T T T T T T T T T T T T T T T T    
669	. . . . C C C C C C C C C C C C C C C C C C    
670	. . . . G G G G G G G G G G G G G G G G G G    
671	. . . . T T T T T T T T T T T T T T T T T T    
672	. . . . G G G G G G G G G G G G G G G G G G    
673	. . . . C C C C C G C C C C C C C C C C C C    
674	. . . . G G G G G . G G G G G G G G G G G G    
675	. . . . T T T T T . T T T T T T T T T T T T    
676	. . . . G G G C C . G G G G G G G G G G G G    
677	. . . . G G G . . . G G G G G G G G G G G G    
678	. . . . C C C . . . C C C C C C C C C C C C    
679	. . . . T T T . . . T T T T T T T T T T T T    
680	. . . . A A A . . . A A A A A A A A A A A A    
681	. . . . G G G . . . G G G G G G G G G G G G    
682	. . . . C C C . . . C C C C C C C C C C C C    
683	. . . . T T T . . . T T T T T T T T T T T T    
684	. . . . C C C . . . C C C C C C C C C C C C    
685	. . . . G G G . . . G G G G G G G G G G G G    
686	. . . . G G G . . . G G G G G G G G G G G G    
687	. . . . G G G . . . G G G G G G G G G G G G    
688	. . . . A A A . . . A A A A A A A A A A A A    
689	. . . . A A C . . . A A A A A A A A A A A A    
690	. . . . C C . . . . C C C C C C C C C C C C    
691	. . . . A . . . . . A A A A A A A A A A A A    
692	. . . . A . . . . . A A A A A A A A A A A A    
693	. . . . C . . . . . C C C C . . C C C C C C    
694	. . . . C . . . . . C C C C . . C C C C C C    
695	. . . . C . . . . . C C C C . . C C C C C C    
696	. . . . T . . . . . T T T T . . T T T T T T    
697	. . . . T . . . . . T T T T . . T T T T T T    
698	. . . . C . . . . . C C C C . . C C C C C C    
699	. . . . T . . . . . T T T T . . T T T T T T    
700	. . . . T . . . . . T T T T . . T T T T T T    
701	. . . . C . . . . . C C C C . . C C C C C C    
702	. . . . C . . . . . C C T T . . T T T T C C    
703	. . . . C . . . . . C C . . . . . . . . C C    
704	. . . . C . . . . . C C . . . . . . . . C C    
705	. . . . C . . . . . C C . . . . . . . . C C    
706	. . . . C . . . . . C C . . . . . . . . C C    
707	. . . . C . . . . . . . . . . . . . . . G G    
708	. . . . . . . . . . . . . . . . . . . . G G    
709	. . . . . . . . . . . . . . . . . . . . C C    
710	. . . . . . . . . . . . . . . . . . . . C C    
711	. . . . . . . . . . . . . . . . . . . . A A    
712	. . . . . . . . . . . . . . . . . . . . T T    
713	. . . . . . . . . . . . . . . . . . . . A A    
714	. . . . . . . . . . . . . . . . . . . . A A    
715	. . . . . . . . . . . . . . . . . . . . T T    
716	. . . . . . . . . . . . . . . . . . . . G G    
717	. . . . . . . . . . . . . . . . . . . . G G    
718	. . . . . . . . . . . . . . . . . . . . C C    
719	. . . . . . . . . . . . . . . . . . . . C C    
720	. . . . . . . . . . . . . . . . . . . . A A    
721	. . . . . . . . . . . . . . . . . . . . C C    
722	. . . . . . . . . . . . . . . . . . . . T T    
723	. . . . . . . . . . . . . . . . . . . . C C    
724	. . . . . . . . . . . . . . . . . . . . T T    
725	. . . . . . . . . . . . . . . . . . . . G G    
726	. . . . . . . . . . . . . . . . . . . . C C    
727	. . . . . . . . . . . . . . . . . . . . G G    
728	. . . . . . . . . . . . . . . . . . . . T T    
729	. . . . . . . . . . . . . . . . . . . . T T    
730	. . . . . . . . . . . . . . . . . . . . G G    
731	. . . . . . . . . . . . . . . . . . . . A A    
732	. . . . . . . . . . . . . . . . . . . . T .    
733	. . . . . . . . . . . . . . . . . . . . A .    
734	. . . . . . . . . . . . . . . . . . . . C .    
735	. . . . . . . . . . . . . . . . . . . . C .    
736	. . . . . . . . . . . . . . . . . . . . A .    
737	. . . . . . . . . . . . . . . . . . . . C .    
738	. . . . . . . . . . . . . . . . . . . . T .    
739	. . . . . . . . . . . . . . . . . . . . G .    
740	. . . . . . . . . . . . . . . . . . . . C .    
741	. . . . . . . . . . . . . . . . . . . . T .    
742	. . . . . . . . . . . . . . . . . . . . T .    
743	. . . . . . . . . . . . . . . . . . . . C .    
744	. . . . . . . . . . . . . . . . . . . . C .    
745	. . . . . . . . . . . . . . . . . . . . C .    
746	. . . . . . . . . . . . . . . . . . . . C .
```
